# A comparative study regarding the adulteration detection of honey: Physicochemical parameters vs. impedimetric data

**DOI:** 10.1016/j.crfs.2023.100642

**Published:** 2023-11-17

**Authors:** Mircea Oroian, Florina Dranca, Sorina Ropciuc, Daniela Pauliuc

**Affiliations:** Faculty of Food Engineering, Stefan cel Mare University of Suceava, Romania

**Keywords:** Honey, Adulteration, Gold impedimetric sensing

## Abstract

Honey adulteration is a major issue for European Union and its members because of an unfair practice of different producers and beekeepers, many adulterations involve the addition of sweet, concentrated syrups which may appear like honey. In our study we analysed the influence of adulteration of tilia honey with different syrups (such as corn, rice, inverted sugar, agave, maple syrups) in different percentages (5%, 10%, and 20% respectively) on physicochemical parameters (moisture content, L*, h_ab,_c_ab_, pH, free acidity, electrical conductivity (EC), 5-hydroxymetilfurfural (HMF), fructose, glucose, sucrose, turanose, trehalose, melesitose and raffinose) and impedimetric properties using electrochemical impedance spectroscopy. The impedimetric sensing was made using an electrochemical cell composed of two gold electrodes, and the frequency ranged between 0.1 kHz and 100 kHz. The impedimetric parameters (Z′, Z″ and phase) and Randal circuit parameters can distinguish the authentic honeys from the adulterated ones (based on the adulteration agent and adulteration percentage, respectively). The partial least squares – discriminant analysis (PLS-DA) and support vector machines (SVM) were used in a binary mode to separate the authentic honeys from the adulterated ones, and the SVM proved to separate much better than PLS-DA.

## Highlights

• The honey adulteration influenced the physicochemical and EIS parameters.
• The EIS was successful in classifying the adulterated samples with concentrated sugar syrups.
• The impedimetric parameters and Randal circuit parameters can distinguish the authentic honeys from the adulterated ones.
• SVM proved to separate much better than PLS-DA the adulterated samples from the authentic ones.

## Graphical abstract

**Figure:**
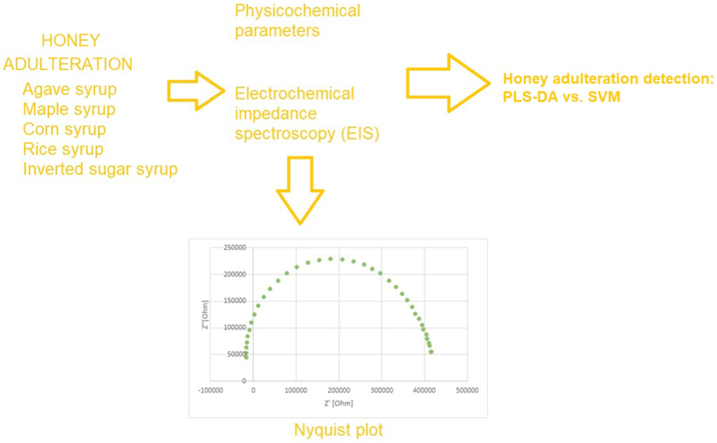

## 1 Introduction

Honey is the most natural sweetener used by human, being produced by bees from the nectar of plants or from sweet secretions of plants. From chemical point of view, it contains fructose, glucose, water, oligosaccharides, and small amounts of amino acids, minerals, vitamins, flavonoids, phenolic compounds, volatile compounds, organic acids (Escriche et al., 2011; Juan-Borrás et al., 2014a, 2014b; Oroian, 2012; Oroian et al., 2015a, b, c; Pauliuc et al., 2020; Pauliuc and Oroian, 2020). The European Union is the second largest producer of honey (275 000 tones) after China (500 000 tones); although UE is a large honey producer, yearly more than 170 000 tones are imported from non-UE countries (worth 405.9 million euros) such as: Ukraine, China, Argentina (Eurostat, 2021). Honey adulteration is a major issue for the authorities; the increasing of honey consumption led to an increase of fraudulent practices worldwide. The OPSON X (Operation OPSON is a Europol INTERPOL joint operation targeting fake and substandard food and beverages) operation ran from December 2020 to June 2021 and showed that 7% of the honey analysed were non-compliant products which means 51 000 kg of fraudulent treated honey. While in a study released by the EU named *From the hives*, 46% samples of a total of 320 samples analysed are suspicious and not complying with the UE directive on honey (*From the Hives, 2023*). In the USP database of adulterated food, honey is placed on the third position (Limm et al., 2023; Moore et al., 2012). In 2019, the Canadian government released a report which showed that 22% of 244 honey analysed were adulterated (*Canadian Government Report: Enhanced Honey Authenticity Surveillance (2018 to 2019)*, 2019), while in a survey conducted on Indian market revealed that all the honey were adulterated (Phipps, 2009). The main adulteration of honeys involves: addition of cheap sweeteners (e.g., sucrose from beet or canes, corn sugar, rice sugar, fructose, high-fructose corn syrups, maltose, inverted sugar), sugar feeding of bees or the mislabeling of the true origin of the product (botanical origin or geographical origin) (Cordella et al., 2002; El-Bialee and Sorour, 2011; Lawal Ridawan A. et al., 2009; Oroian et al., 2017, 2019; Piotr et al., 2016; Siddiqui et al., 2017a, 2017b).

Honey adulteration detection methods involves high performance liquid chromatography (HPLC) (Du et al., 2015), gas chromatography (GC) (Arroyo-Manzanares et al., 2019; Plutowska et al., 2011), nuclear magnetic resonance (NMR) (Cagliani et al., 2022; Ribeiro et al., 2014), stable carbon isotope ratio analysis (SCIRA) (Daniele et al., 2012; Kawashima et al., 2019), Fourier transform infrared spectroscopy (Anjos et al., 2015; F. Huang et al., 2020), Raman spectroscopy (Oroian et al., 2017) and electronic tongue (Ciursa and Oroian, 2021b). Although the methods presented above present high sensitivity, precision an accuracy, many of them are expensive, are not ecofriendly or laborious, and the obtained data is difficult to be interpreted for routine purposes (Wang et al., 2019; Xu et al., 2019).

Electrochemical impedance spectroscopy (EIS) is a rapid, simple, cost-effective, non-destructive and high-throughput electrochemical technique which was used for characterization of food products (T. K. Huang et al., 2021; Minetto et al., 2022). This technique consists in applying an alternating current or voltage signal (with a small sinusoidal perturbation at fixed frequency) to an electrochemical cell (e.g. 2–4 electrodes) which contains the investigated matrix; the alternating current or voltage signal response at the same frequency is measured and transformed into impedance (T. K. Huang et al., 2021; Minetto et al., 2022; Soares et al., 2020). The Nyquist plot (where for each frequency, real part of impedance (Z’) is plotted versus imaginary part of impedance (Z”)) is obtained by applying the process described above to different frequencies (Aguedo et al., 2020; T. K. Huang et al., 2021). EIS was applied for the characterization of vegetable tissues (Ando et al., 2016) and beverages (Soares et al., 2020), for identifying of raw bovine milk adulteration with urea (Minetto et al., 2022), milk characterization (Lopes et al., 2018), determination of electrical parameters of honey (Paszkowski et al., 2014), determination of fish freshness (Sun et al., 2018). Therefore, this study aimed to evaluate the application of EIS to identify the intentional addition of different adulteration agents (corn, rice, inverted sugar, agave and maple syrups) to honey. To our knowledge this technique was not used for the detection of honey adulteration with the syrups studied in the present study.

## 2 Materials and methods

### 2.1 Honey samples

22 samples of tilia honeys were purchased from local beekeepers from Suceava county, Romania, from the 2022 season. The samples were authenticated according to the melissopalynological method (Louveaux et al., 1978) (all samples had more than 60% of pollen grains from *Tilia europaea*). They were kept at 4 °C prior analysis.

### 2.2 Honey adulteration

From the 22 sunflower honey samples, 5 authentic honeys (2 samples with low percentage of *Tilia europaea* pollen grains – 62.1% and 63.2%, 1 sample with medium percentage of *Tilia europaea* pollen grains – 65.2%, respectively, and 2 samples with high percentage of *Tilia europaea* pollen grains 68.9% and 70.8%) were adulterated with different percentages of agave, maple, corn, rice, and inverted sugar syrups (from beet sugar) in different percentages (5%, 10% and 20%, respectively).

### 2.3 Physicochemical parameters determination

The International Honey Commission methods were used for the determination of pH, free acidity, moisture content and electrical conductivity (Bogdanov, 2002). The color was determined in CIEL*a*b* coordinates using a CR-400 chromameter (Konica Minolta, Japan). The 5-hydroxymethylfurfural content was determined by the spectrophotometric method published by White (1979). High performance liquid chromatography (HPLC) was used to determine the sugar content (fructose, glucose, sucrose, turanose, trehalose, melesitose and raffinose) according to the method proposed by Bogdanov (2002).

#### 2.3.1 EIS measurement

Two gold electrodes (2.0 mm in diameter) placed at 4 mm one to the other were introduced directly in the honey (20 g). The EIS measurement was carried out from 0.1 kHz to 100 kHz with an unbiased 100 mV sinusoidal signal using a PGSTAT 204 with FRA32M module (Metrohm, Filderstadt, Germany). The data were recorded and analysed with NOVA 2.0 software (Metrohm, Filderstadt, Germany). All analyzes were performed in triplicate, at 25 °C degree.

The EIS is a powerful technique for the characterization of electrochemical systems, and in particular on the characteristics of food products which contain certain substances dissolved (e.g. ions) which may conduct electricity. The impedance of system (Z) is calculated using the Ohm's law, and in Cartesian coordinates is given by:

$$Z=Z^{\prime }-j∙Z^{\prime \prime }$$

where Z′ is the real part of the impedance, Z″ is the imaginary part, and $j=\sqrt{-1}$.

### 2.4 Statistical analysis

The results were submitted to analysis of variance (ANOVA) and Pearson correlation using XLSTAT trial version (Microsoft, Charlotte, NC, USA). Fisher's least significant difference (LSD) procedure was used at the 95% confidence level.

The partial least squares-discriminant analysis (PLS-DA) model and support vector machines (SVM) model were performed using XLSTAT trial version (Microsoft, USA). To attain precise classification of authentic and adulterated samples, various key parameters, including accuracy, sensitivity (which assesses the correct identification of true positives), and specificity (which assesses the correct identification of true negatives), were measured. These parameters were calculated using the following equations (Barbosa et al., 2016):

(1) $$Accuracy=\frac{True\;positives+True\;negatives}{True\;positives+True\;negatives+False\;positives+False\;negatives}$$

(2) $$Sensitivity=\frac{True\;positives}{True\;positives+False\;negatives}$$

(3) $$Specificity=\frac{True\;negatives}{True\;negatives+False\;positives}$$

The data were split into two categories: calibration set (66.6% of the samples) and validation set (33.3% of the samples); the samples were chosen randomly by the software.

## 3 Results and discussions

### 3.1 Physicochemical properties

In Table 1 are presented the physicochemical properties of tilia honeys analysed (mean, minimum, maximum, and standard deviations). The tilia honeys moisture content ranged between 17.0 and 19.9, below the limit established by the European Union at 20% (Eu Council, n.d.), the level was in agreement with the literature (Abera and Alemu, 2023; Oroian, 2015); a moisture content higher than 20% can depreciate the honey because the fermentation process which may occur.

**Table 1:** Physicochemical parameters of tilia honey (n = 22).

Variable	Minimum	Maximum	Mean	Std. deviation
L*	34.3	41.3	38.2	1.7
**h**_**ab**_	87.6	92.2	90.0	1.0
C*_ab_	26.7	35.7	31.9	2.6
pH	4.1	5.6	5.0	0.4
**Free acidity (meq acid/100g)**	1.0	12.6	2.7	2.1
**EC (μS/cm)**	537.0	763.0	567.2	122.5
**Moisture (%)**	17.0	19.9	18.6	1.0
**HMF (mg/kg)**	0.4	26.1	6.0	6.2
**Fructose (%)**	31.9	35.1	33.0	0.8
**Glucose (%)**	26.8	32.0	28.7	1.5
**Sucrose (%)**	0.1	2.2	0.4	0.7
**Melesitose (%)**	0.0	2.4	1.1	0.7
**F/G**	1.1	1.2	1.2	0.1

The color is the main parameter which influences the perception of consumer toward a certain food, and it is described by: lightness L* (white/dark), h*_ab_ (hue – represents the perceived color of an object) and C*_ab_ (chroma – describes the intensity, saturation or purity of a color, high values belonging to rich colors and low values to grey colors) (Tuberoso et al., 2014). As Table 1 shows, the L* parameter value ranged between 34.3 and 42.3 with a mean value of 38.2, the h_ab_ ranged between 87.6 and 92.2, while the C*_ab_ ranged between 26.7 and 35.7. The values were in agreement with other studies made on tilia honeys (Juan-Borrás et al., 2014a; Pauliuc et al., 2021).

The electrical conductivity is influenced by the level of minerals dissolved in and by the presence of organic acids in honeys (Kivrak et al., 2017a, b). The electrical conductivity ranged between 337.0 and 763.00 μS cm^−1^ (Table 1) values which were closed to those reported honey tilia honeys from Spain, Czech Republic, and Italy (Juan-Borrás et al., 2014a; Persano Oddo and Piro, 2004).

The pH and the free acidity of honey are correlated with the extraction and storage of it, and the level of them may influence the shelf life of the product (Kivrak et al., 2017b). The pH values ranged between 4.1 and 5.6 (Table 1), while the free acidity ranged between 1.0 and 12.6 meq acid/100 g, which is below the level set by UE (Eu Council, n.d.).

Hydroxymethylfurfural (HMF) is a quality criterion for honey and occurs due to the chemical reactions which may be formed when the product is kept at unsuitable temperature or the heat treatment is inadequate (Shapla et al., 2018). The HMF ranged between 0.4 and 26.1 mg/kg honey (Table 1), below the level of 40 mg/kg established for honey (Eu Council, n.d.), the values of the HMF are in the agreement with the literature (Besir et al., 2021; Grainger et al., 2017, 2017n, 2017ret al., 2018).

The individual sugar profile of each honey may be used for the botanical authentication, and they have an impact on the physicochemical properties (e.g., osmolarity), and the fructose/glucose ratio influence the crystallization process (Islam et al., 2022). The main sugar presented is fructose (31.9–35.1%), followed by glucose (26.8–32.0%) and sucrose (0.1–2.2%). The ratio fructose/glucose (F/G) is between 1.1 and 1.2, and for this reason it has a crystallize tendency shortly after extraction. The melesitose ranged between 0.0 and 2.4%. Turanose, trehalose, and raffinose were below the limit of quantification. All the data can be found in Table 1.

#### 3.1.1 Influence of adulteration agents on physicochemical parameters

In Table 2 are presented the addition of different percentages (5, 10 and 20%) of sugars syrups into honey (maple, agave, rice, corn, and inverted sugars). The L*, fructose and glucose content decreased significantly with the addition of maple syrup addition into honey, the electrical conductivity and sucrose content increased significantly with the addition of the maple syrup. The moisture content was not influenced significantly by the addition of maple syrup but at concentration higher than 10%, the level was higher than 20% (the threshold established by the UE). The electrical conductivity, glucose content, decreased significantly with the addition of agave syrup, while HMF, fructose content, F/G increased significantly. L*, the fructose content, glucose content, sucrose content and F/G decreased significantly with the addition of rice syrup, while the free acidity and melesitose increased significantly, respectively. EC, fructose content, glucose content decreased significantly with the addition of corn syrup. The inverted sugar addition influenced the EC which decreased significantly, while the HMF increased significantly, the addition of more than 10% syrup lead to a concentration higher than the level established by the UE (the inverted sugar has a high concentration of HMF because of the heat treatment applied during the sucrose conversion (Ciursa and Oroian, 2021a)). From all the 5 syrups used for the adulteration, the maple syrup generated the most intensive changes (e.g. L*, EC, glucose, sucrose).

**Table 2:** Physicochemical parameters variation in function of adulteration with maple, agave, rice, corn, and inverted syrups.

Variable	Maple addition (%)				F	Agave addition (%)				F	Rice addition (%)				F	Corn addition (%)				F	Inverted sugar (%)				F
	0	5	10	20		0	5	10	20		0	5	10	20		0	5	10	20		0	5	10	20	
L*	39.92^a^	39.04^a^	38.15^a^	36.37^b^	22.5***	39.92^a^	40.21^a^	40.51^a^	41.08^a^	2.43 ^ns^	39.92^abc^	39.29^bc^	38.66^d^	37.41^d^	11.33**	39.92^abc^	40.51^bc^	41.09^cd^	42.26^d^	9.81**	39.92^a^	40.03^a^	40.14^a^	40.36^a^	0.35 ^ns^
hab	89.42^a^	89.44^a^	89.48^a^	89.54^a^	0.01 ^ns^	89.42^a^	89.34^a^	89.28^a^	89.15^a^	0.05 ^ns^	89.42^a^	89.46^a^	89.52^a^	89.62^a^	0.03 ^ns^	89.42^a^	89.44^a^	89.47^a^	89.53^a^	0.01 ^ns^	89.41^a^	89.52^a^	89.63^a^	89.54^a^	0.15 ^ns^
cab	31.95^a^	30.66^a^	29.36^a^	26.78^a^	1.11 ^ns^	31.95^a^	31.92^a^	31.87^a^	31.79^a^	0.01 ^ns^	31.95^a^	31.30^a^	30.66^a^	29.36^a^	0.28 ^ns^	31.95^a^	30.71^a^	29.47^a^	26.99^a^	1.02 ^ns^	31.95^a^	31.87^a^	31.78^a^	31.61^a^	0.01 ^ns^
pH	5.46^a^	5.53^a^	5.56^a^	5.73^a^	3.11 ^ns^	5.46^a^	5.40^a^	5.36^a^	5.22^a^	2.51 ^ns^	5.46^a^	5.42^a^	5.38^a^	5.31^a^	1.01 ^ns^	5.46^a^	5.42^a^	5.37^a^	5.28^a^	1.53 ^ns^	5.46^a^	5.38^a^	5.29^a^	5.11^a^	5.65 ^ns^
Free acidity (meq acid/100 g)	2.73^a^	2.76^a^	2.78^a^	2.83^a^	0.01 ^ns^	2.73^a^	2.76^a^	2.78^a^	2.82^a^	0.05 ^ns^	2.73^c^	3.69^bc^	4.66^abc^	6.58^ab^	4.24*	2.73^a^	2.77^a^	2.80^a^	2.87^a^	0.01 ^ns^	2.73^a^	2.92^a^	3.10^a^	3.47^a^	0.15 ^ns^
EC (μS/cm)	664.6^c^	677.0^abc^	709.3^ab^	774.0^a^	7.7**	664.6^a^	614.08^ab^	583.4^bc^	522.32^cd^	6.93*	644.6^a^	645.3^a^	645.9^a^	647.2^a^	0.03 ^ns^	644.6^ab^	613.2^ab^	581.8^b^	519.1^bc^	7.31*	644.6^ab^	617.1^ab^	589.5^ab^	534.3^bc^	5.64*
Moisture (%)	19.21^a^	19.87^a^	20.54^a^	21.87^a^	1.4 ^ns^	19.21^a^	19.39^a^	19.58^a^	19.96^a^	0.12 ^ns^	19.21^a^	19.35^a^	19.51^a^	19.81^a^	0.07 ^ns^	19.21^a^	19.12^a^	19.03^a^	18.86^a^	0.02 ^ns^	19.21^a^	19.14^a^	19.08^a^	18.95^a^	0.02 ^ns^
HMF (mg/kg)	3.24^a^	3.26^a^	3.27^a^	3.31^a^	0.01 ^ns^	3.24^d^	9.00^c^	14.76^b^	26.27^a^	86.4***	3.24^a^	4.45^a^	5.67^a^	8.10^a^	3.85 ^ns^	3.24^a^	3.22^a^	3.20^a^	3.16^a^	0.01 ^ns^	3.24^a^	36.19^b^	69.1^c^	135.1^d^	2831***
Fructose (%)	32.45^a^	30.94^ab^	29.42^bc^	26.38^d^	74.8***	32.45^c^	33.21^bc^	33.97^bc^	35.50^a^	18.9***	32.45^a^	30.84^ab^	29.23^c^	26.01^d^	84.3***	32.45^a^	30.83^ab^	29.21^c^	25.97^d^	85.5***	32.45^a^	32.57^a^	32.70^a^	32.95^a^	0.5 ^ns^
Glucose (%)	27.69^ab^	26.46^abc^	25.22^bc^	22.75^d^	82.7***	27.69^ab^	27.14^ab^	26.58^abc^	25.45^bc^	16.7***	27.69^a^	27.04^ab^	26.39^c^	25.09^d^	22.91***	27.69^a^	26.38^ab^	25.08^c^	22.46^d^	92.5***	27.69^a^	27.87^a^	28.04^a^	28.42^a^	1.71 ^ns^
Sucrose (%)	0.21^d^	3.04^c^	5.87^b^	11.53^a^	2202***	0.21^d^	0.23^a^	0.24^a^	0.27^a^	0.05 ^ns^	0.22^a^	0.22^a^	0.21^a^	0.20^a^	0.01 ^ns^	0.21^a^	0.21^a^	0.20^a^	0.18^a^	0.03 ^ns^	0.22^a^	0.22^a^	0.22^a^	0.22^a^	0.0 ^ns^
Melesitose (%)	1.11^a^	1.05^a^	0.99^a^	0.89^a^	0.02 ^ns^	1.11^a^	1.05^a^	0.99^a^	0.88^a^	0.02 ^ns^	1.11^d^	2.87^c^	4.65^b^	8.19^a^	26.75***	1.11^d^	2.82^c^	4.53^b^	7.96^a^	25.02***	1.10^a^	1.06^a^	0.99^a^	0.89^a^	0.02 ^ns^
F/G	1.17^a^	1.17^a^	1.17^a^	1.16^a^	0.19^ns^	1.17^c^	1.22^c^	1.28^b^	1.39^a^	65.1***	1.17^a^	1.14^ab^	1.11^bc^	1.04^d^	26.51***	1.17^a^	1.17^a^	1.17^a^	1.16^a^	0.32 ^ns^	1.17^a^	1.17^a^	1.17^a^	1.16^a^	0.21 ^ns^

^ns^-not significant *P > 0.05*, * *- P < 0.05*, ** - *P < 0.01*, *** - *P < 0.001*.

#### 3.1.2 Honey EIS properties

**Fig. 1:**
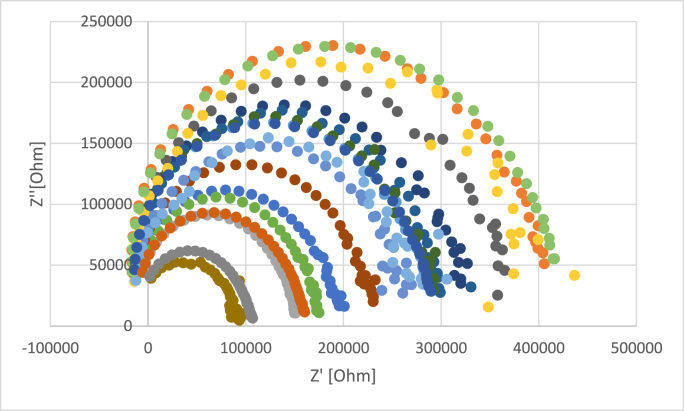
Nyquist plot for tilia honeys (each color represents a different sample)

**Fig. 2:**
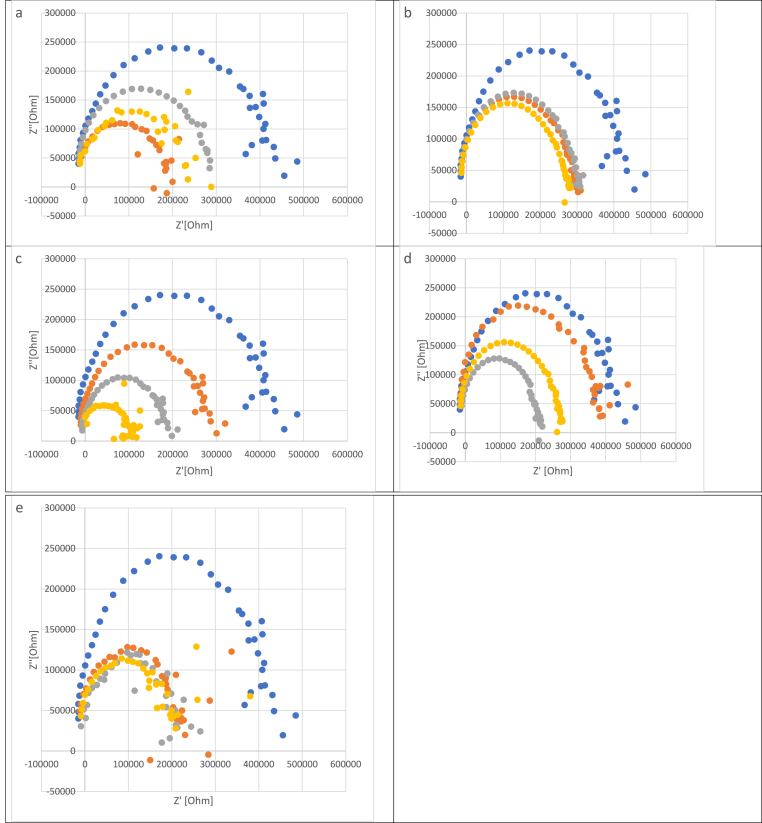
Nyquist plot for tilia honey (a sample with medium percentage of *Tilia europaea* pollen grains – 65.2%) adulterated with: a-agave, b-inverted sugar, c-maple, d-corn, e-rice, blue circle – honey, orange circle – 5% adulteration, grey circle – 10% adulteration, yellow circle – 20% adulteration

**Fig. 3:**
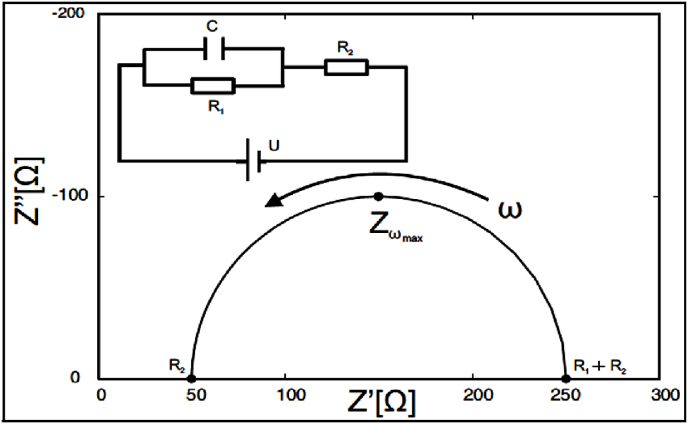
Randles circuit and application to Nyquist plot: solution resistance (R_S -_R_2_), double layer capacitance (CD - C) and charge transfer resistance (R_CT_ -R_1_).

The projection of the real part of the impedance vs. the imaginary part of the impedance is called Nyquist plot. In Fig. 1 is presented a typical Nyquist plot for tilia honey samples (the frequency ranged between 0.1 KHz and 100 kHz); the electrochemical active species presented into honeys are phenols, vitamins are prone to generate an exchange current at electrode interface by means of a small potential. Honey contains a large spectrum of organic acids (e.g., gluconic, formic, lactic, acetic, citric, malic acids) (Pauliuc and Oroian, 2020); these compounds can act as functioning as supporting electrolytes which may diminish the Ohmic drop phenomenon and inhibit the migration of electroactive substances toward the polarized electrodes, ultimately leading to an augmentation in electrochemical current density (Tsierkezos and Ritter, 2012). The differences between the Nyquist plot of the tilia sample is because of the electrical conductivity of the samples (EC = 537–763 μS/cm), which influence the electrical circuit. In Fig. 2 is presented the Nyquist plot for the honey (the sample with medium percentage of *Tilia europaea* pollen grains – 65.2%) adulterated with agave, inverted sugar, maple, corn, and rice syrups in different concentrations (5%, 10% and 20%). As can be observed in Fig. 2 the addition of adulteration agents led to a decrease of the circle radius of Nyquist plot. To achieve a fitting of the data, the experimental impedance spectra data were subjected to a conventional Randles equivalent circuit model (depicted in Fig. 3), encompassing components such as solution resistance (R_S_), double layer capacitance (CD) and charge transfer resistance (R_CT_). In Table 3 are presented the data about the impedance, phase and Randles model parameters; the data are presented in two ways: the first one authentic vs. adulterated samples, and the second one authentic vs. each adulteration agent and its addition percentage. When we compare the authentic honeys vs. adulterated ones, there can be observed high differences with respect to Z′, Z″, phase, R_s_ and R_1._ The solution resistances of the adulterated are much higher because the content of electrochemically active species and supporting electrolytes are much lower (T. K. Huang et al., 2021). In the case of each adulteration agent, there can be observed that in the case of maple syrups addition, the increase of the adulteration agent led to a decrease of the R_s_ in opposition with the behaviour of the other adulterations, this fact maybe attributed to the electrochemical species presented (the electrical conductivity of the samples with maple syrups are much higher (Table 2)). The Warburg impedance decreased with the increasing of the adulteration agents. The R_s_, CD and R_CT_ of the samples analysed are different from the literature because the electrical conductivity of the authentic honey taken for analysis were much lower (T. K. Huang et al., 2021).

**Table 3:** Effect of adulteration on the honey impedance properties.

		Log Z’^a^	Log Z”^a^	Phase (°)^a^	Randle circuit parameters		
					R_s_ (kΩ)	CD·(nF)	R_CT_ (kΩ)
**Honey (n** = **22 samples)**		5.35^a^	5.11^a^	27.72^a^	24.4^b^	0.027^a^	269.3^a^
**Adulterated (75 samples)**		5.23^b^	4.87^b^	20.96^b^	26.7^a^	0.028^a^	190.4^b^
**Corn**	**0**	5.35^c^	5.11^a^	27.72^a^	24.4^d^	0.027^a^	269.3^a^
	**5**	5.36^c^	4.95^ab^	22.85^ab^	25.4^c^	0.027^a^	225.3^b^
	**10**	5.40^b^	4.92^b^	19.62^b^	27.7^b^	0.030^a^	181.5^b^
	**20**	5.48^a^	4.91^b^	13.71^c^	29.1^a^	0.026^a^	224.3^b^
**Agave**	**0**	5.35^a^	5.11^a^	27.72^a^	24.4^a^	0.027 ^a^	269.3^a^
	**5**	5.23^b^	4.88^b^	21.63^b^	25.4^b^	0.027 ^a^	206.1^b^
	**10**	5.22^b^	4.84^bc^	21.20^c^	26.1^b^	0.027 ^a^	204.1^b^
	**20**	5.21^b^	4.74^c^	20.59^d^	27.9^a^	0.027 ^a^	186.5^c^
**Rice**	**0**	5.35^a^	5.11^a^	27.72^a^	24.4^c^	0.027 ^a^	269.3^a^
	**5**	5.20^b^	4.81^b^	22.10^b^	24.5^bc^	0.030 ^a^	180.4^b^
	**10**	5.17^b^	4.82^b^	25.84^b^	27.1^b^	0.046 ^a^	164.5^c^
	**20**	5.15^c^	4.86^b^	24.18^b^	29.5^a^	0,030 ^a^	153.9^d^
**Inverted sugar**	**0**	5.35^a^	5.11^a^	27.72^a^	24.4^d^	0.027 ^a^	269.3^a^
	**5**	5.26^b^	4.87^b^	20.91^b^	25.1^c^	0.027 ^a^	206.8^b^
	**10**	5.24^b^	4.82^b^	20.10^b^	26.3^b^	0.027 ^a^	205.2^b^
	**20**	5.21^c^	4.78^bc^	18.38^c^	26.6^a^	0.026 ^a^	207.7^b^
**Maple**	**0**	5.35^a^	5.11^a^	27.72^a^	24.4^a^	0.027 ^a^	269.3^a^
	**5**	5.18^b^	4.74^b^	16.21^b^	22.9^b^	0.016 ^a^	239.3^b^
	**10**	5.09^c^	4.64^c^	14.72^c^	21.1^c^	0.029 ^a^	125.6^c^
	**20**	4.93^d^	4.42^d^	12.27^d^	20.5^d^	0.027 ^a^	103.3^d^

a at f = 10 MHz, solution resistance (R_S_), double layer capacitance (CD), charge transfer resistance (R_CT_), and Warburg impedance (W).

The group comparisons among authentic honeys and adulterated honeys were made using ANOVA with Fisher's least significant difference (LSD) procedure at 95% confidence level; there were observed a significant difference (P < 0.05) between the parameter's studies presented in Table 3 (double layer capacitance have not a clear evolution with the adulteration agent addition). The addition of 5% adulteration agents (agave, inverted sugar, rice and corn sugars) has significantly influenced the parameters presented in Table 3.

The Pearson correlation reveled that the physicochemical parameters are well correlated with the impedimetric parameters, such as Z′ with pH (r = −0.360**), Z″ with pH (r = −0.438*), phase with pH (r = −0.423**), R_CT_ with pH (r = −0.430**), Z′ with electrical conductivity (r = −0.288*), Z″ with electrical conductivity (r = −0.258*), phase with electrical conductivity (r = −0.243*), fructose with R_CT_ (r = 0.319**), glucose with Z’‘ (r = 0.344**), phase (r = 0.371**) and R_CT_ (r = 0.372**), sucrose with Z′ (r = −0.401**), Z’‘ (r = −0.287*), phase (−0.343**) and R_CT_ (r = −0.338**), Z’ and melesitose (r = 0.279*). The meaning of * is that the correlation is significant at the 0.05 level (2-tailed), and ** is that the correlation is significant at the 0.01 level (2-tailed).

#### 3.1.3 Honey adulteration detection

Honey adulteration detection involved the utilization of two statistical methodologies: partial least squares – discriminant analysis (PLS-DA) and support vector machines (SVM). The two models were applied to: (a) physicochemical data (the parameters from Table 1), (b) impedimetric data (Z′, Z″ and phase) and (c) physicochemical + impedimetric data, in order to discriminate the authentic honeys from the adulteration ones, and also the classification of authentic honeys from each adulteration type (agave, maple, corn, inverted sugar, and rice sugars).

#### 3.1.4 Honey adulteration detection - classification of pure honey and adulterated honeys using PLS-DA and SVM

The PLS-DA and SVM models were checked to see which of the two statistical analyses can better distinguish the samples using the physicochemical data, impedimetric data and physicochemical + impedimetric data. It was taken into consideration the accuracy, sensitivity and specificity as indicators for selecting the best analyses. In Table 4 are presented the statistical analysis results for PLS-DA and SVM.

**Table 4:** Binary classification of pure honey and adulterated honeys using PLS-DA and SVM based on (a) physicochemical data, (b) impedimetric data and (c) physicochemical + impedimetric data.

		Physicochemical data			Impedimetric data			Physicochemical + impedimetric data		
		Accuracy (%)	Sensitivity (%)	Specificity (%)	Accuracy (%)	Sensitivity (%)	Specificity (%)	Accuracy (%)	Sensitivity (%)	Specificity (%)
Authentic vs. adulterated										
PLS-DA	Calibration	93.18	75.00	100	77.27	50	90	93.18	78.57	100
	Validation	91.30	80.00	100	69.56	25.0	93.33	86.95	75.00	93.33
SVM	Calibration	100	100	100	100	100	100	100	100	100
	Validation	95.65	88.88	100	100	100	100	100	100	100
Authentic vs. adulterated with agave syrup										
PLS-DA	Calibration	95.23	100	75	71.42	93.33	16.66	95.23	100	80
	Validation	80	100	60	80	58.71	66.66	100	100	100
SVM	Calibration	100	100	100	100	100	100	100	100	100
	Validation	90.00	90.00	100	100	100	100	100	100	100
Authentic vs. adulterated with maple syrup										
PLS-DA	Calibration	95.23	100	80	85.71	93.75	60	100	100	100
	Validation	90	100	75.00	70.00	100	25.00	80	100	50
SVM	Calibration	100	100	100	100	100	100	100	100	100
	Validation	100	100	100	100	100	100	100	100	100
Authentic vs. adulterated with corn syrup										
PLS-DA	Calibration	95.23	100	85.71	66.66	85.71	28.57	100	100	100
	Validation	100	100	100	70.00	87.50	0	80	100	50
SVM	Calibration	100	100	100	100	100	100	100	100	100
	Validation	100	100	100	100	100	100	100	100	100
Authentic vs. adulterated with rice syrup										
PLS-DA	Calibration	100	100	100	76.19	86.66	50	100	100	100
	Validation	80	100	50	90.00	100	66.67	80	100	50
SVM	Calibration	100	100	100	100	100	100	100	100	100
	Validation	70	70	70	100	100	100	100	100	100
Authentic vs. adulterated with inverted sugar syrup										
PLS-DA	Calibration	100	100	100	66.66	85.71	28.57	95.23	100	85.71
	Validation	90	100	75	80	87.50	50.00	90	100	50
SVM	Calibration	100	100	100	100	100	100	100	100	100
	Validation	100	100	100	100	100	100	100	100	100

The PLS-DA models reached lower parameters for the discrimination of the samples (authentic vs. adulterated) than the SVM models; in some cases, the accuracy, sensitivity and specificity of the SVM models were 100%, much higher than in the PLS-DA models.

Based on the input data, the PLS-DA models built on physicochemical data had statistical parameters much better than on the models built on impedimetric data, while the models built on physicochemical + impedimetric data had the best statistical parameters. The SVM models built using the impedimetric data yielded superior statistical outcomes compared models built with physicochemical data.

Honey adulteration detection-Classification of pure honey (0% adulteration) and each concentration of adulteration agent (5%, 10% and 20%) using PLS-DA and SVM based on EEM spectra.

The adulteration detection in this part was focused on the classification of the authentic honeys from the adulterated ones in function of the adulteration percentage (0% vs 5%, 0% vs 10%, 0% vs 20%). The PLS-DA and SVM were checked to see which of the two statistical analyses can distinguish better the samples using (a) physicochemical data, (b) impedimetric data and (c) physicochemical + impedimetric data. It was taken into consideration the accuracy, sensitivity and specificity for choosing the best analyze. In Table 5are presented the statistical analysis results for PLS-DA and SVM.

**Table 5:** **Binary classification of pure honey (0% adulteration) and each concentration of adulteration (5%, 10% and 20%) using PLS-DA and SVM based on** a) physicochemical data, (b) impedimetric data and (c) physicochemical + impedimetric data.

		Physicochemical data			Impedimetric data			Physicochemical + impedimetric data		
		Accuracy (%)	Sensitivity (%)	Specificity (%)	Accuracy (%)	Sensitivity (%)	Specificity (%)	Accuracy (%)	Sensitivity (%)	Specificity (%)
0% vs 5%										
PLS-DA	Calibration	95.83	92.85	100	70.83	71.42	70.00	100	100	100
	Validation	62.30	80.50	100	61.53	75.00	40.00	74.64	60.00	100
SVM	Calibration	100	100	100	100	100	100	100	100	100
	Validation	100	100	100	100	100	100	100	100	100
0% vs 10%										
PLS-DA	Calibration	100	100	100	70.83	87.50	37.50	100	100	100
	Validation	69.23	85.71	100	69.26	100	42.85	84.64	71.42	100
SVM	Calibration	100	100	100	100	100	100	100	100	100
	Validation	100	100	100	100	100	100	100	100	100
0% vs 20%										
PLS-DA	Calibration	100	100	100	95.83	100	88.89	100	100	100
	Validation	100	100	100	84.61	100	66.66	84.61	100	100
SVM	Calibration	100	100	100	100	100	100	100	100	100
	Validation	100	100	100	100	100	100	100	100	100

The PLS-DA models reached lower parameters for the discrimination of the samples (authentic vs. adulterated with 5%/10%/20%) than the SVM models; in some cases, the accuracy, sensitivity and specificity of the SVM models were 100%, much higher than in the PLS-DA models. Based on the input data, the PLS-DA models built on physicochemical data had statistical parameters much better than on the models built on impedimetric data, while the models built on physicochemical + impedimetric data had the best statistical parameters. The SVM models built using the impedimetric data yielded superior statistical outcomes compared models built with physicochemical data. It is important to mention that it was observed an increase of the statistical parameters of the PLS-DA model with the increase of the adulteration agent addition (Table 5).

## 4 Conclusions

In this study was observed the influence of adulteration of tilia honeys with different percentages (5%, 10% and 20% respectively) of syrups (rice, corn, agave, maple, and inverted sugar) on physicochemical parameters and impedimetric parameters. It can be observed that the adulteration with maple increased the moisture content over the threshold established by the European Union, while the honey adulterated with inverted sugar (more than 10%) had a concentration of HMF over 40 mg/kg. The fructose, glucose and sucrose profile changed in the case of honey adulterated with maple, corn, rice, and agave syrups, while in the case of inverted sugar adulteration the profile was not modified significantly. The impedimetric parameters (Z′, Z″ and phase) and Randal circuit parameters can distinguish the authentic honeys from the adulterated ones (based on the adulteration agent and adulteration percentage, respectively). There can be observed a high negative correlation between pH and Z′, Z″, phase and R_CT_, electrical conductivity and Z′ and Z″, and positively correlation between glucose and Z″, phase and R_CT_. In binary classification tasks using physicochemical data, impedimetric data, and a combination of physicochemical and impedimetric data, support vector machines (SVM) outperformed partial least squares – discriminant analysis (PLS-DA) in distinguishing authentic honey from adulterated samples for each type of adulteration agent. Specifically, when classifying pure honey (0% adulteration) against various concentrations of adulteration agent (5%, 10%, and 20%), SVM achieved superior statistical results compared to PLS-DA. This study confirms the utility of a simple method that can be used for *in situ* adulteration detection of honey with a different syrup used worldwide. The EIS measurement has a real potential of determining *on-situ* the compounds which are correlated with impedimetric properties (e.g. (e.g., gluconic, formic, lactic, acetic, citric, malic acids).

## CRediT authorship contribution statement

**Mircea Oroian:** Validation, Formal analysis, Data curation, Writing – original draft, Visualization, All authors have read and agreed to the published version of the manuscript. **Florina Dranca:** and, and, Writing – review & editing, and. **Sorina Ropciuc:** Software, Methodology, and, Resources, Supervision. **Daniela Pauliuc:** and, Investigation, and.

## Declaration of competing interest

The authors declare the following financial interests/personal relationships which may be considered as potential competing interests:

Mircea Oroian reports financial support was provided by 10.13039/100020629Stefan cel Mare University of Suceava. Mircea Oroian reports a relationship with Universitatea Stefan cel Mare din Suceava that includes: employment. Mircea Oroian has patent pending to No applicable. not applicable.

## Data Availability

No data was used for the research described in the article.
